# Top electrode materials for semi-transparent perovskite solar cells: A review

**DOI:** 10.1007/s42452-025-07883-5

**Published:** 2025-11-07

**Authors:** Ram Datt, Hind Alsayyed, Shivani Dhall, Sonal Gupta, Swati Bishnoi, Ramashankar Gupta, Sandeep Arya, Trystan Watson, Wing Chung Tsoi

**Affiliations:** 1 Faculty of Science and Engineering, SPECIFIC, Swansea University, Bay Campus, Fabian Way, Swansea, SA1 8EN UK; 2 Department of Physics, DAV College, Jalandhar, 144008 Punjab India; 3 Department of Chemistry and The Institute for Lasers, Photonics, and Biophotonics, University at Buffalo, SUNY, Buffalo, New York 14260 USA; 4 Department of Chemical Engineering, Indian Institute of Technology, Delhi, New Delhi, 110016 India; 5 Advanced Materials and Metrology Division, CSIR-National Physical Laboratory, New Delhi, 110012 India; 6 Department of Physics, University of Jammu, Jammu, 180006 Jammu and Kashmir India

**Keywords:** Transparent top electrode, Semi-transparent solar cells, Perovskite solar cells

## Abstract

The rising demand for renewable energy solutions has accelerated interest in semi-transparent solar cells (STSCs) for emerging applications such as building-integrated photovoltaic, automotive systems, and wearable electronics. Perovskite solar cells (PSCs) show considerable promise as STSCs due to their high performance, cost-effectiveness, solution processability, compatibility with flexible substrates, and transparency of perovskite films. Collaborative efforts have been directed towards developing transparent top electrodes (TTEs) and device architectures for PSCs to enhance the performance and transparency. The choice of top electrode materials significantly influences the performance and transparency of semi-transparent perovskite solar cells (STPSCs). Various materials such as dielectric/metal/dielectric (DMD) layers, metal thin film, metal nanowires, transparent conducting oxide (TCO), conductive polymers (e.g., PEDOT: PSS), graphene, and carbon nanotubes have been identified as potential TTEs. TCO, DMD, and metal thin film electrodes typically require sputtering or thermal deposition methods; others are solution-processable. The material selection and thickness of the top electrode play crucial roles in improving both the efficiency and transparency of PSC devices, posing challenges in optimising device performance while maintaining high transparency. This review comprehensively covers the essential material characteristics required for top electrodes in STPSCs; surveys reported top electrode materials and discusses their characterisation, stability, scalability, current challenges, and prospects.

## Introduction

 Perovskite solar cells (PSCs) have garnered significant attention due to their remarkable potential for high efficiency and low-cost fabrication. Perovskite, named after its structure resembling the naturally occurring mineral perovskite, utilise a hybrid organic-inorganic perovskite material as the light-absorbing layer for preparing the PSCs devices. The unique properties of perovskite, such as high charge carrier mobility, tunable bandgap, and facile solution processing, make it particularly promising for solar cells application [1]. In recent years, power conversion efficiency (PCE) for PSCs has grown dramatically, reaching 26.7% and 30.1% for single and tandem (perovskite-perovskite) structure devices [2–4]. As a result, solar cells made of perovskite are being regarded as a strong contender to replace conventional silicon-based solar cells, which hold a substantial share of the solar cells market. PSCs have already demonstrated their potential for aerospace and indoor applications, yielding benefits from when applied as semi-transparent solar cells (STSCs) [5]. The chemical formula for perovskite is ABX₃, where A represents a monovalent cation (such as methylammonium, formamidinium, or caesium), B represents divalent metals (such as Pb^2+^ or Sn^2+^), and X represents a halide anion (such as Cl⁻, I⁻, or Br⁻) [6, 7]. Perovskites are distinguished by their remarkable optical and electrical features, including a direct and adjustable energy gap, large charge carrier diffusion length, and a high absorption coefficient [8, 9]. Their fabrication is compatible with solution-based and evaporation techniques, allowing for many simple deposition processes, including evaporation, spin coating, slot-die, roll-to-roll, and screen printing [10–12]. 

Perovskite’s appealing features allow for the fabrication of semi-transparent perovskite solar cells (STPSCs), in which some light is transmitted through the cells. At the same time, the rest gets absorbed by the perovskite photoactive absorber layer. Preparing a thin layer of perovskite material and by partially covering the perovskite layer can achieve the desired transparency. Transparency has also been tuned by manipulating the band gap of the perovskite materials [13]. Buildings and skyscrapers with semi-transparent solar cell windows can provide shading and generate renewable energy [14]. In addition, semi-transparency of PSC’s makes them well-suited for applications in greenhouse structures, automobile windows, residential and commercial windows, power-generating bus stands, and other similar infrastructures [15, 16]. By generating power independently of the electrical grid, constructing integrated photovoltaic systems may significantly reduce greenhouse gas emissions and building energy usage. However, STPSCs may be incorporated into the sides of a building to optimise space capacity and energy output. At the same time, most traditional solar cells are often situated on roofs and in open areas for optimum exposure to sunlight. In PSCs, the thickness of the photoactive perovskite layers typically ranges from 150 to 1000 nm, and a layer with a thickness of 200 nm is sufficient to produce transparency while still maintaining device performance [17, 18]. The electrodes represent the device’s terminal component, which could influence its transparency. Materials such as aluminium-doped zinc oxide (AZO), fluorine-doped tin oxide (FTO), and indium tin oxide (ITO) are frequently employed as bottom (front) electrodes in PSCs devices due to their transparency in the visible and near-infrared regions. In contrast, the top (back) electrode plays a dominant role in maintaining the solar cell’s transparency and has thus become an essential component for developing STPSCs. The optical and electrical properties of the top electrodes are essential for selecting the material if device transparency and performance are to be preserved. Also, due to the demand for displays and optoelectronics devices, the need for transparent, conductive, and flexible electrodes has surged over the past two decades [19]. Consequently, there has been an extensive study of transparent conducting oxides (TCOs). ITO has seen considerable application as a transparent electrode in displays and at the front side of PSCs. Transparent top electrodes (TTEs) in STPSCs are the subject of intensive study due to the need for transparency. Top electrodes need to meet several criteria, including scalable manufacturing, cost-effectiveness, compatibility with the underlying layer, affordability, transparency, and high conductivity. The fabrication of solar cells generally includes the use of gold (Au), aluminium (Al), copper (Cu), and silver (Ag) electrodes (typically 100 nm thick). Most STPSCs have utilised a metal thin layer (10–20 nm), carbon nanotubes, metal nanowires, graphene, etc [20–22]. Their deposition or coating method also impact the electrode transparency.

While numerous studies have reviewed electrode materials for commercialising PSCs, comprising PSCs for highly transparent and semi-transparent applications, the critical role of the top electrode in determining transparency has often been overlooked [23–25]. In this review, we have highlighted the significance of top electrodes in STPSCs and presented a comprehensive overview of the current state-of-the-art in top electrode materials. This review compiles a summary of the literature on top electrodes for STPSCs, encompassing the materials used, the methods of deposition, and the results obtained. Additionally, we provide an in-depth description of the characterisation of the top electrodes. Moreover, we have discussed stability and scalability, along with existing difficulties and the need for potential developments.

## Transparent top electrodes (TTEs) in STPSCs

**Fig. 1:**
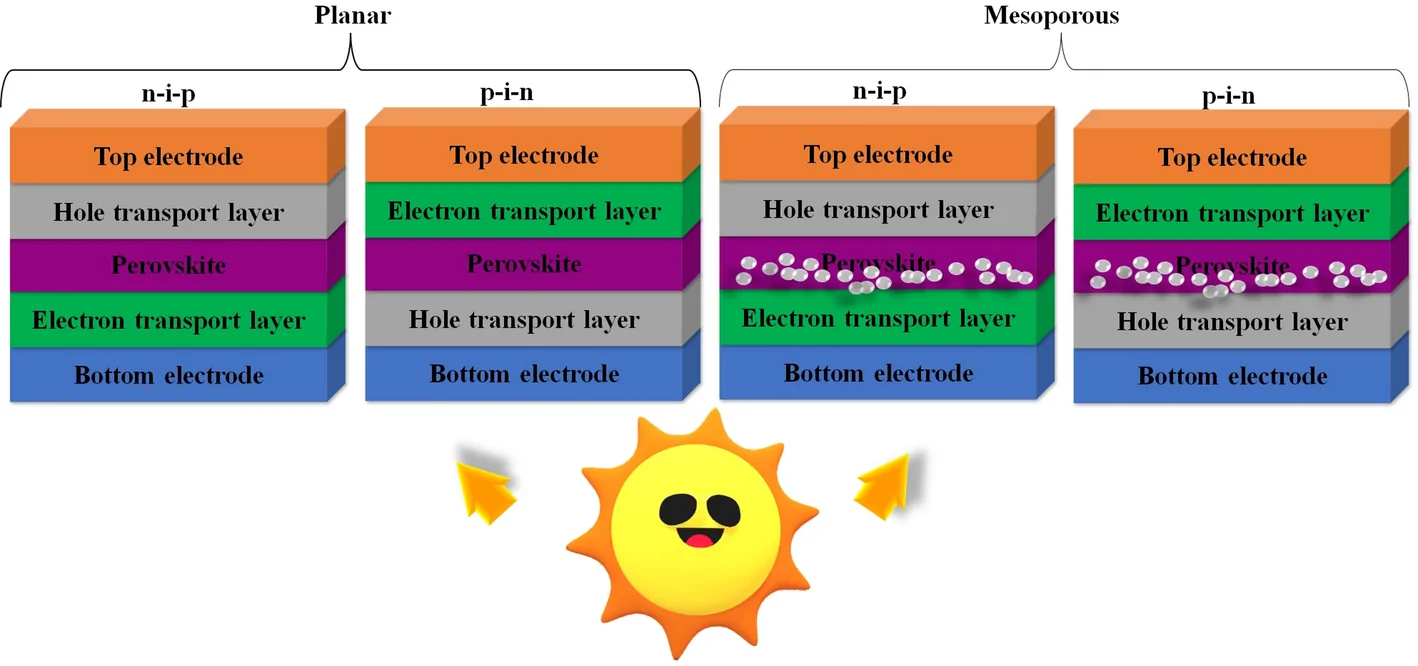
The device structure of PSCs: planar, and mesoporous

The two categories of PSCs are fabricated, involving planar and mesoporous structures. Figure 1 demonstrates the architecture of the device, the arrangement and the sequence of the functional layers within the device. The top electrode is opaque and comes last during fabrication. It influences device transparency, charge carrier collection, PCE, stability, and scalability of solar cells. The STPSCs device maintained semi-transparency using a thin metal layer, metal nanowires (NWs), and graphene electrodes [26, 27]. As transparent electrodes, a metal oxide and dielectric/metal/dielectric (DMD) material structure has also been implemented. Gaspera et al. [28] presented a DMD for transparent top contact, and it was made up of a layer of MoO_3_ on the bottom, a layer of Au in the middle, followed by MoO_3_ layer on the top. The bottom MoO_3_ layer serves as a seeding layer, which enables the creation of a continuous layer by allowing for improved surface coverage of the thin Au film. The top MoO_3_ layer guards the reflectance of the metal and determines the transmission window of the DMD structure. When a thin Au layer was deposited directly on top of the charge transport layer, it showed high series resistance (4.3 × 10^3^ Ω cm^2^). The conductivity improved dramatically after a very thin (1 nm) MoO_3_ layer was placed before the Au layer. However, if the MoO_3_ layer becomes thick (20 nm), then it limits the amount of charge carrier extraction and decreases the device efficiency. It is also feasible to boost the conductivity of the contact by depositing a thicker Au coating for greater percolation. This would, however, reduce the device’s overall transparency.

The essential attributes for efficient TTEs include favourable work function, conductivity, transparency, scalability, stability, and low sheet resistance. Specific characteristics are inherently interrelated; for example, increasing film thickness improves conductivity but diminishes transparency. Consequently, there exists a perpetual trade-off between electrode transparency and conductivity.

**Fig. 2:**
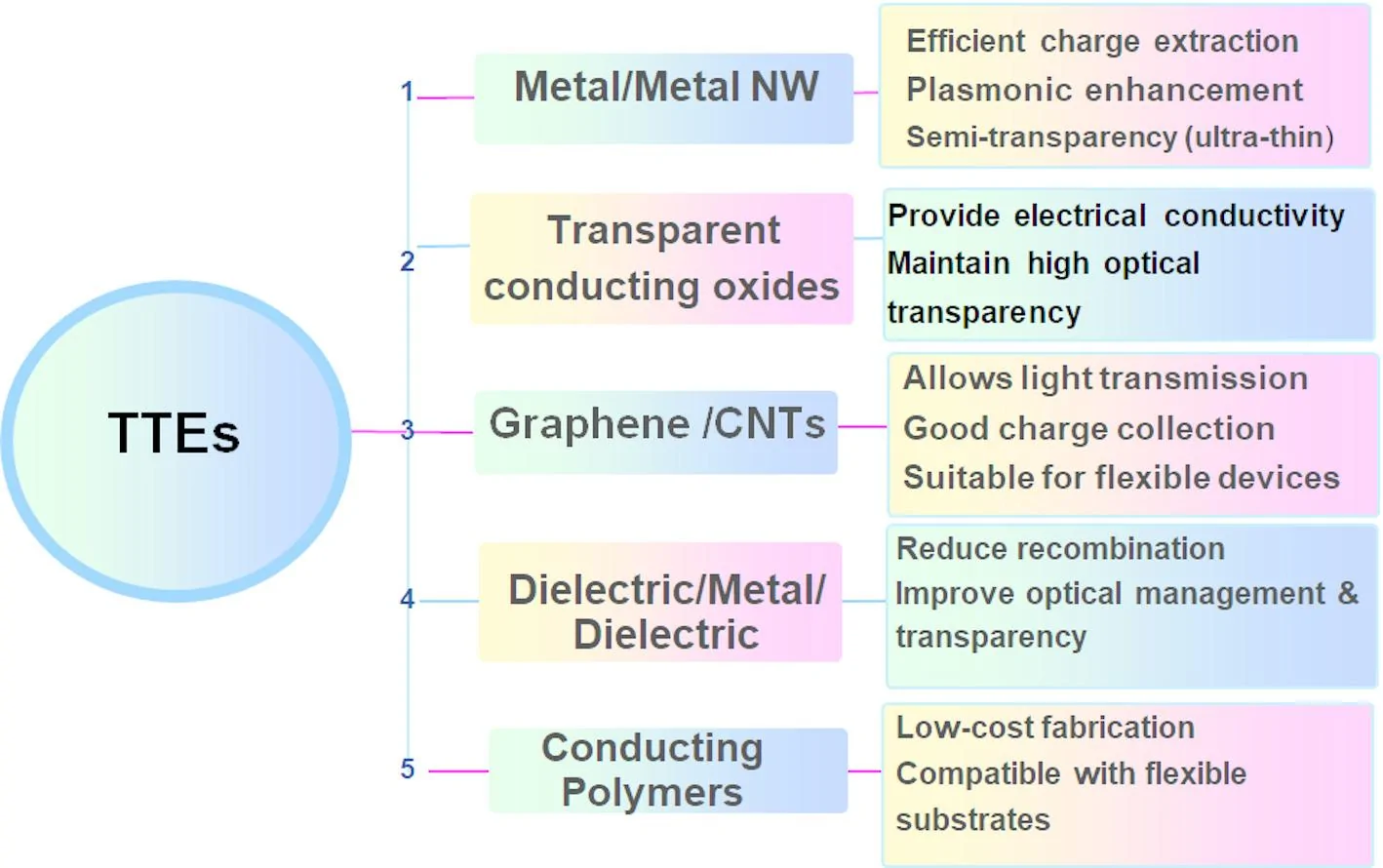
Classification of transparent top electrode materials used in STPSCs and their key advantages

The typical metal electrode materials, such as Al, Ag, and Au, have been thoroughly implemented as transparent electrodes and were deposited as a thin layer (16–20 nm). An ITO, the most well-known transparent TCO, has also been examined as a potential candidate for use in TTEs. Compared to metallic electrodes, TCOs have greater resistivity and a more brittle coating that lacks appropriate chemical stability. In addition, the DMD multilayer frameworks and TCOs have also been used in PSCs to function as the top electrode. Their vacuum deposition process and concerns relating to a low degree of transparency have caused researchers to redirect their emphasis toward developing alternative materials. Carbon nanotubes (CNTs), graphene, metal nanowires NWs, conducting polymers, etc., are solution-processable materials, and their advantages are shown in Fig. 2. The following sections present a summary of top electrode materials and their utilisation in PSCs.

### TTE coating techniques impact

The top electrode material thickness and deposition parameters impact the electrode transparency and conductivity. Sun et al. [29] optimised the thickness and the deposition rate of the metal top electrode. The Ag film with a thickness of 10 nm was deposited on top of the MoO_3_ film (10 nm) at different deposition rates, viz. 0.2, 0.5, 1.0, and 1.5 Å/s and the change in deposition rate shows a change in transparency in the film. The 1.0 and 1.5 Å/s deposition rates provided encouraging results, with increased transmittance across the visible area. The average visible transmittance (AVT) and the sheet resistance go down when the thickness of the Ag film increases. The impact of deposition rate on the silver film was investigated using scanning electron microscopy (SEM). Microstructure images revealed that a low deposition rate of 0.2 Å/s led to the formation of isolated islands, resulting in irregular morphology. These discontinuous islands hindered charge transfer, leading to high sheet resistance. Conversely, higher deposition rates of 1.0 Å/s and 1.5 Å/s promoted agglomeration across the film, creating a continuous channel between islands for efficient transmission of charge carriers.

**Fig. 3:**
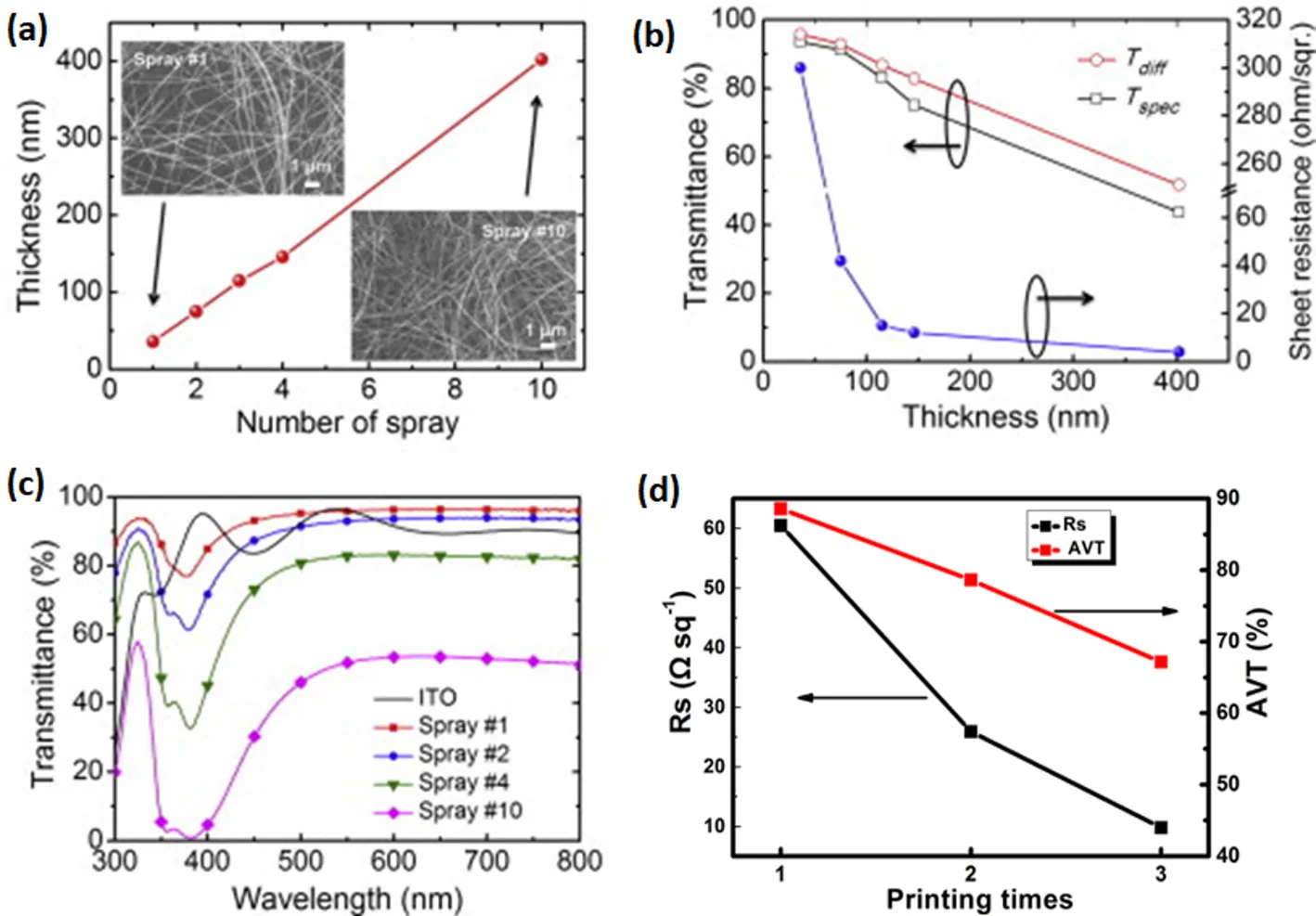
(**a**) Variation of thickness of spray-coated Ag NWs with number of spray times (inserted SEM image of spay 1 and 10 coated film), (**b**) Spray-coated Ag NW electrode’s diffusive transmittance (T_diff_), specular transmittance (T_spec_) and sheet resistance, (**c**) transmittance (Diffusive) spectra of Ag NW electrodes coated at different spray [30]. (Reproduced with permission) Copyright © 2014 Elsevier B.V. All rights reserved. (**d**) Variation of AVT and sheet resistance as a function of printing times of inkjet coated Ag NWs [31]. (Reproduced with permission) Copyright © 2018 WILEY-VCH Verlag GmbH & Co. KGaA, Weinheim

In the realm of spray-coated films, the number of sprays determines the coated film’s thickness and its conductivity. Kang et al. [30] reported on spray-coated Ag nanowires, adjusting the film thickness from 50 to 400 nm through 1–10 spray applications, as depicted in Fig. 3(a). The sheet resistance fluctuated between 300 and 4 Ω sq⁻¹, with transmittance ranging from 95% to 51% for film thicknesses of 50 and 400 nm, respectively, as shown in Fig. 3 (b). Optimisation of the spray-coated film is essential to achieve optimal outcomes. Similarly, the number of inkjet printing cycles influences the characteristics of the top electrode. Initial printing of the thin film often yields high sheet resistance and AVT. However, increasing the number of printing cycles can decrease electrical sheet resistance and transmittance (Fig. 3c). According to Xie et al. [31] the sheet resistance of inkjet-printed Ag nanowires showed a decreasing trend with an increase in printing cycles and a reduction in AVT (Fig. 3(d)).

## TTE materials for STPSCs

**Fig. 4:**
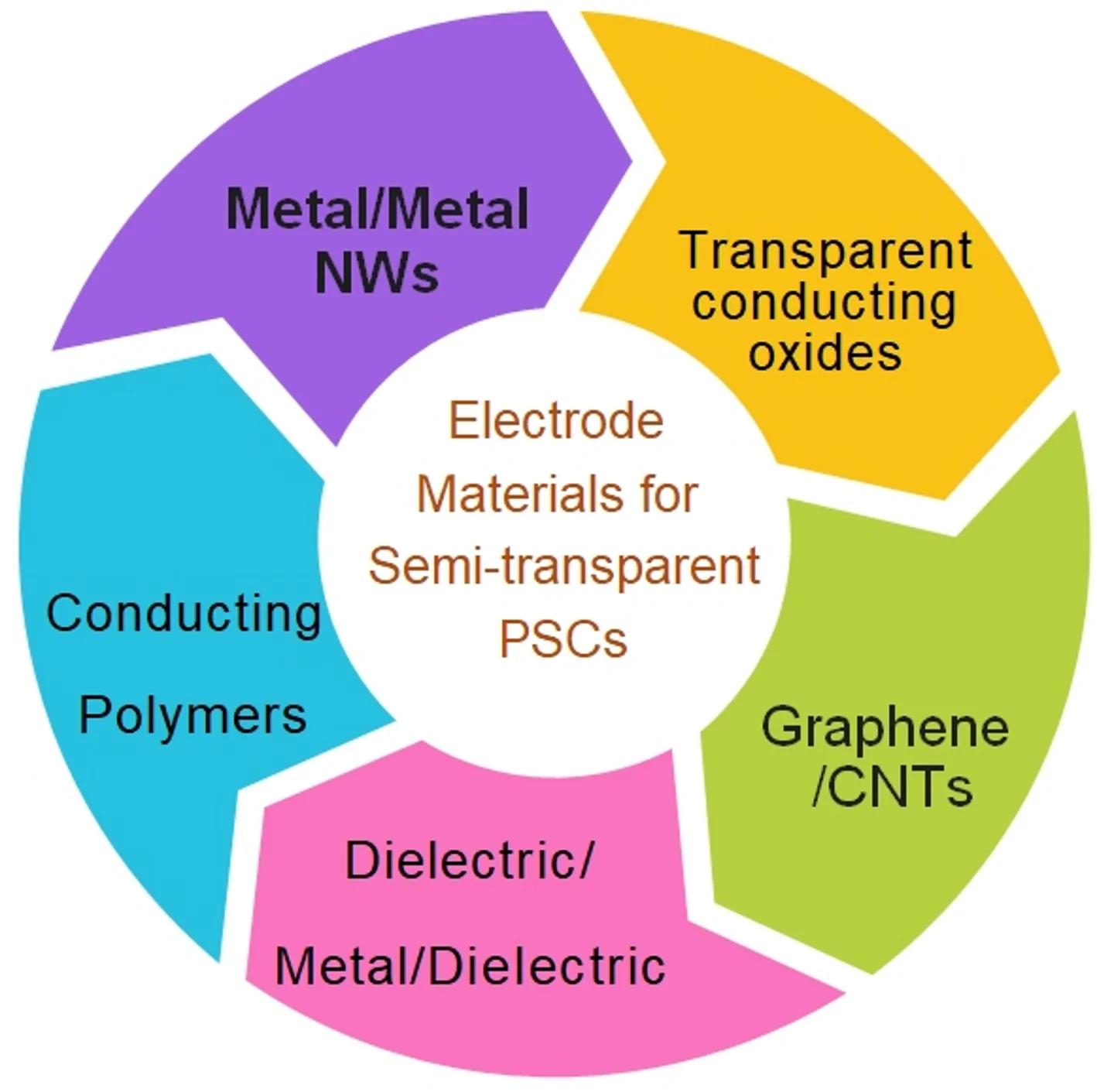
Overview of top electrode material categories used in STPSCs

Organic-inorganic lead halide PSCs are emerging as significant contenders in the solar cell market, with their PCE reached similar to Silicon solar cells within a decade. Recently, they have shown promise as STSCs. Various techniques have been reported to manipulate the device’s transparency, including tuning the perovskite film’s bandgap and coating the thin film [13, 32]. The choice of top electrodes profoundly impacts the device’s transparency. As shown in the Fig. 4, numerous materials such as thin metal films, metal nanowires, conducting polymers, graphene, and CNTs have been explored for adding top electrode transparency. Table 1 is showing the AVT and PCEs of various photoactive layers and top electrode materials reported in literature.

### Metal and metal nanowires (NW) based TTEs

**Fig. 5:**
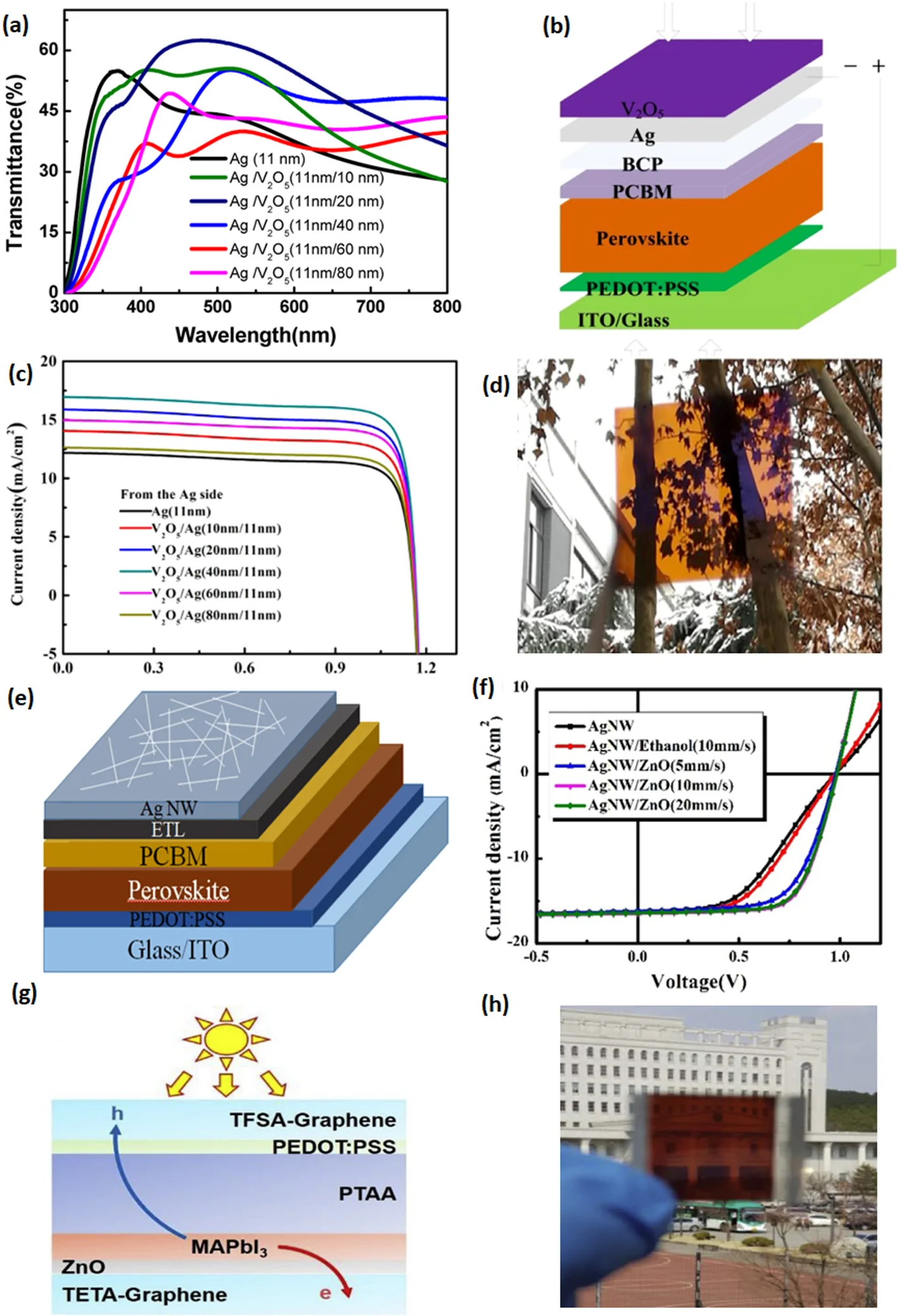
(**a**) Optical transmittance of Ag/glass and V_2_O_5_/Ag/Glass films, (**b**) device geometry of bifacial PSCs, (**c**) J-V curves of bifacial PSCs, and (**d**) photograph of bifacial PSCs with V_2_O_5_ as top optical coupling layer [35]. Reproduced with permission. Copyright © 2018 American Chemical Society. (**e**) PSCs device structure with spray coated top AgNWs, (**f**) J-V characteristics of Ag NWs and Ag NW/ZnO electrode-based PSCs device [38]. Reproduced with permission. Copyright © 2018 Elsevier B.V. All rights reserved. (**g**) Schematic device geometry of flexible and transparent PSCs, (**h**) photograph of flexible (TETA-Graphene top electrode) and STPSC device [39]. Reproduced with permission. Copyright © 2018 Elsevier B.V. All rights reserved

Metals and metal nanowires as transparent top electrodes have been explored in the literature. In 2014, Eperon et al. [33] reported planar standard (FTO/c-TiO_2_/perovskite/spiro-OMeTAD/Au) PSCs, and the device showed PCE up to 3.5% with the AVT of ~ 30%, by applying a thin layer of Au (10 nm) as a TTE. Roldán-Carmona et al. [34] demonstrated an Au (6 nm)/LiF (100 nm) top electrode for an STPSCs device (ITO/PEDOT: PSS/ CH_3_NH_3_PBI_3_ / Au/ LiF). The optimized device delivered PCE up to 6.4% with AVT of ~ 29%. This implementation is based on perovskite thin film (40 nm), deposited by thermal-evaporation technique. The LiF layer protects the thin gold layer and reduces the loss of specular reflection energy. The LiF layer is termed as capping layer. Pang et al. [35] fabricated bifacial semi-transparent inverted planar structured PSCs, which employed Ag thin film top electrode and V_2_O_5_ as an optical coupling layer. The bifacial PSCs can absorb light from both the top and bottom sides. A device with an Ag thin film electrode (bare Ag) exhibited a PCE of 14.62% by illumination from the ITO side and 5.45% PCE on illumination from the Ag side. After introducing the V_2_O_5_ (20 nm) optical coupling layer on top of Ag, the transparency of the corresponding electrodes (Fig. 5(a)), as well as the PCE of the device, has been enhanced (up to ~ 8.91%) from Ag side illumination. The enhancement in PCE was caused by the increase in short-circuit current density (J_SC_) while open-circuit voltage (V_OC_) and fill factor (FF) were approximately similar. On the other hand, the PCE was almost identical for ITO side illumination (PCE = 14.01%). In the wavelength range of 300 nm to 800 nm, the bare Ag thin film (11 nm) has an AVT of 38.49%. The AVT 48.44% is reported when the thickness of the V_2_O_5_ layer was 20 nm. Using the thicker V_2_O_5_, AVT begins to decrease. When the thickness of the V_2_O_5_ reaches 60 nm, the AVT (36.76%) of the Ag/V_2_O_5_ drops compared to bare Ag thin film (38.49% AVT). The corresponding device architecture and current density-voltage (J-V) characteristics when illuminated from the Ag side are shown in Fig. 5(b, c). Figure 5(d) shows a picture of the PSCs device with the V_2_O_5_ optical coupling layer. The study revealed the Ag/V_2_O_5_ double layer could be used as a potential semi-transparent electrode, and it pointed out a new direction for the bifacial PSCs. Przypis et al. [36] applied thin Ag (10 nm) electrode in flexible substrate-based device structure of PET/IZO/PEDOT: PSS/perovskite/C_60_/BCP/Ag STPSCs. The devices delivered PCE (AVT) of 5.70% (21.5%) and 1.90% (41.1%) using 30 and 10 nm thick perovskite film, respectively.

Guo et al. [37] showed efficient STPSCs (ITO/ PEDOT: PSS / Perovskite/ PC_60_BM / Ag NW) using solution-processable Ag NWs as the top electrode, and the device yielded a PCE of 8.49% and control device (vacuum-deposited Ag) with opaque electrode was 9.19%. The solution-processed Ag NWs TTE exhibited 5 times higher series resistance compared to the opaque counterpart, which resulted in decreased PCE in the STPSC. Additionally, since the Ag NW electrode has a high transparency, the resulting STPSCs demonstrated a AVT of 28.4%.

Sun et al. [40] demonstrated that mixing a small amount of deionized (DI) water into the Ag NWs dispersion in isopropanol (IPA) improved conductivity and transparency of Ag NWs spray-coated film. The author claimed that water can wash polyvinyl pyrrolidone (PVP) away from the Ag NWs surface and leads to Ag NW bundles. The water has a relatively slow evaporation rate, and there remains high water content during the drying. This residual water washed away the insulating PVP from the Ag NWs surface and promoted the formation of Ag NWs bundles, which helped improve the conductivity. Whereas it is reported that the water solvent for Ag NWs solution can seriously limit the device’s shelf life by harming the perovskite layer underneath [41]. Yang et al. [42] reported a STPSCs (FTO/TiO_2_/MAPbI_3_/Spiro-MeOTAD/Ag NWs) fabricated by all-solution processing route and a spray-coating technique used to deposit Ag NWs electrode. The STPSCs showed maximum PCE of 7.98% using 750 nm Ag NWs and a minimum PCE of 4.56% using 200 nm Ag NWs when illuminated from FTO side. It was found that at 800 nm, the device transmits roughly 67% light without the Ag NWs electrode. Transmission drops substantially from ~ 55 to 35% when Ag NW thickness is increased from 200 to 750 nm. A denser and thicker Ag NWs layer aids in collecting more charges transferred from, and the thicker and denser Ag NWs layer reflects more sunlight into the perovskite absorber, increasing its absorption. It was suggested that the spray-coated Ag NWs electrode not only minimises the production cost but also results in a semi-transparent device, which can be incorporated into building windows as well as a top cell on tandem solar cells. Similarly, a research group fabricated a STPSCs, using spray deposited Ag NWs and ZnO (Ag NW/ZnO) as a top electrode. The spray-coated Ag NWs were found to be weakly connected, leading to poor conductivity [38]. Therefore, an additional layer of ZnO nanoparticles was spray-coated as device structure shown in Fig. 5(e), which formed an Ag NW/ZnO composite electrode. The ZnO nanoparticles filled the voids between Ag NWs and were attributed to the interconnection between Ag NW. They helped improve the Ag NW/ZnO composite conductivity. The composite electrode strategy enhanced the PCE of the device from 7.31 to 11.13% and maintained a high AVT (~ 23%). Figure 5(f) (J-V characteristics) shows the impact of the ZnO layer with Ag NWs. It is shown that the sheet resistance of the Ag NW/ZnO decreased significantly by improving the interconnection of Ag NWs.

### Transparent conducting oxides (TCOs) based TTEs

The transparent conductive oxides (TCOs) are widely used materials in solar cells, due to their conductivity and transparency properties. Mosty, they applied as bottom transparent electrodes and recently, they also explored as TTEs. Werner et al. presented indium zinc oxide (IZO) as a TTE in glass/FTO/c-TiO_2_/m-TiO_2_/CH_3_NH_3_PbI_3_/Spiro-MeOTAD/MoO_x_/electrode PSCs device structure [43]. The devices delivered PCE of 10.36% using IZO, which was lower than control Au electrode (PCE ~ 12.5%) based devices. Ying et el. also reported the IZO as a TTE for preparing the STPSCs, and the devices shown a PCE of 16.14%, which was further improved to 18.19% by introducing BCP: Ag complex before IZO [44]. The introduction of BCP: Ag complex between electron transport layer and IZO suppress charge recombination and protect the underneath layer from the damage caused by the process. Kim et al. sputtered amorphous InZnSnO (IZTO) as TTE to prepare STPSCs, and the devices delivered PCE of 9.081% with the AVT of 22.17% [45]. The device performance was comparable to Ag electrode-based control devices, whereas IZTO electrode added semitransparency. A similar research group reported the two-step linear facing target sputtering (FTS) deposited IZTO, and the electrode named as TS-IZTO, which delivered the PCE of 15.52% while maintaining the AVT of 20.88% [46]. Luca et al. applied the magnetron sputtering deposited ITO as a TTE for making STPSCs [47]. The reported device structure ITO/Spiro-TTB/ perovskite/ C_60_/BCP/ Electrode delivered PCE of 17.9% using sputtered ITO electrode and 18.0% for opaque.

### Graphene and carbon nanotubes based TTEs

Graphene and carbon nanotubes have demonstrated conductive and transparent properties, making them extensively researched candidates for bottom and top electrodes materials in PSCs [48–53]. Jang et al. [39] reported the doping of graphene with bis (trifluoromethanesulfonyl) -amide (TFSA) and triethylenetetramine (TETA) molecules. They were applied as bottom and top electrodes, respectively, in STPSCs, shown in Fig. 5(g). The device delivered a PCE up to 10.56% and AVT of 5%. Figure 5(h) shows the fabricated flexible STPSCs device. Single walled carbon nanotubes (SWCNTs) are well studied in various fields due to their tremendous electrical, mechanical, thermal, and optical properties, and recently they have been applied as TTE for STPSCs fabrication. Sakaguchi et al. [54] demonstrated the SWCNTs as TTEs in FTO/TiO_2_/MAPbI_3_/SWCNTs device. The metal-free device without HTL delivered PCE up to 10.0%, which is caused by the composite functioning of active layer/SWCNT. After coating polymethylmethacrylate (PMMA) on the top of SWCNT’s top electrode, the device’s PCE improved up to 11.8%. The performance was boosted due to the improved densifying effect of SWCNTs network by coating PMMA, which increased the conductivity of SWCNT film. PMMA also worked as an effective encapsulation and demonstrated improved stability in the ambient environment. The device with PMMA coating retained ∼72% of its initial PCE even after 3 weeks, while the device without PMMA coating retained only ∼32% of its initial efficiency. Yoon et al. [55] demonstrated the CNT electrode in all-inorganic PSCs. Interestingly, apart from comparable PCE to metal electrode (Au), the CNT-electrode-based devices maintained their performance for over 4000 h (un-encapsulated in air). Author suggested the formation of robust nanohybrid film by CNT electrode with underneath film (HTL), which boosts the overall device stability. Rehman et al. demonstrated a HTL free carbon electrode based large-area STPSCs modules fabricated using manual screen-printing method [56]. The modules achieved PCE of 12.09 and 11.8% for 100 cm^2^ and 900 cm² module size.

### Dielectric/Metal/Dielectric (DMD) based TTEs

**Fig. 6:**
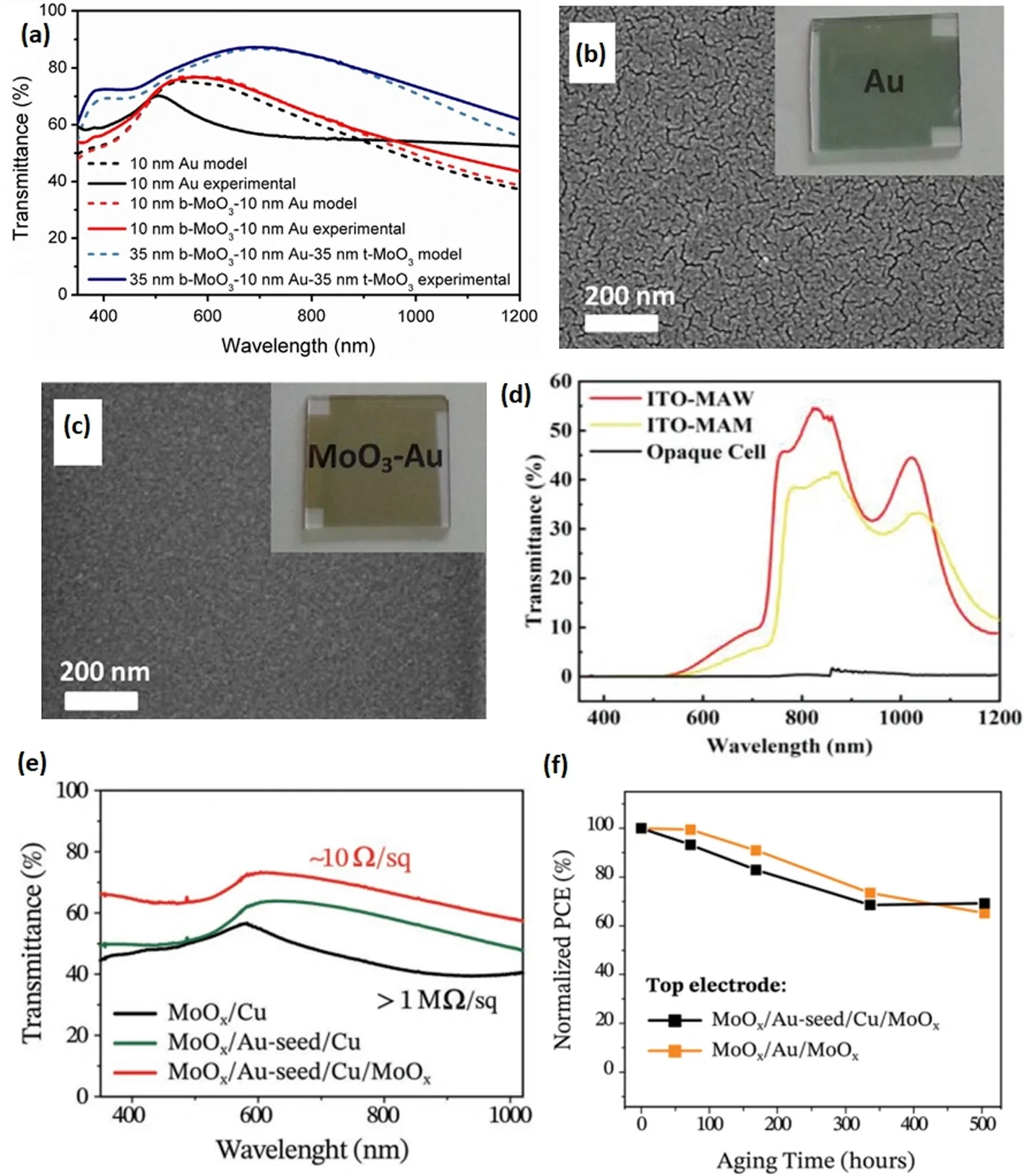
(**a**) Experimental (solid lines) and simulated (dashed lines) transmittance spectra of Au, MoO_3_/Au, and MoO_3_/Au/MoO_3_ top electrodes, (**b**) Au film SEM image, (**c**) MoO_3_/Au film SEM image [28]. Reproduced with permission. Copyright © 2015 Elsevier Ltd. All rights reserved. (**d**) Transmittance of different top electrodes [62]. Reproduced with permission. Copyright © 2020 Wiley-VCH GmbH, (**e**) transmittance spectra of MoO_x_ (6 nm)/Au-seed (1.5 nm)/Cu (11 nm)/MoO_x_ (20 nm), MoO_x_ (6 nm)/Au-seed (1.5 nm)/Cu (11 nm), and MoO_x_ (6 nm)/Cu (11 nm) multilayer structure (sheet resistance also shown), (**f**) the degradation performance of MoO_x_/Au-seed/Cu/MoO_x_ and MoO_x_/Au/MoO_x_ top electrode-based PSCs device [63]. Reproduced with permission. Copyright © 2019 WILEY-VCH Verlag GmbH & Co. KGaA, Weinheim

Due to their transparency and conductivity properties, the DMD multilayer electrodes have been widely employed in STPSCs [57]. It is also called oxide/metal/oxide structure, a thin metal film used to improve the conductivity, whereas oxide film used for optical impedance matching and environmental protection. The structure is reported to achieve low sheet resistance and high optical transmittance. In 2005, Gaspera et al. [28] demonstrated the MoO_3_/Au/MoO_3_(MAuM) multilayer DMD structure as a TTE, and the optimised device (FTO/TiO_2_/CH_3_NH_3_PbI_3_/Spiro-OMeTAD/MAuM) delivered PCE up to 5.3% with 31% AVT. The transmittance spectra (Fig. 6(a)) of Au, MoO_3_/Au, and MAuM have been studied using experimental and software simulation methods. It shows an improvement in transparency compared to bare Au and MoO_3_/Au electrodes. After coating the Au film on the top of glass or Spiro-OMETAD, the Au nucleated into separate islands and possessed localized surface plasmon resonance (LSPR) effect, which resulted in overall transmittance reduction. In contrast, when Au film is deposited over the MoO_3_ thin (1–2 nm) film, it forms a smooth layer. The corresponding SEM images as shown in Fig. 6(b, c) showed that Au-coated film over MoO_3_ formed a uniform and crack-free morphology. The Au deposited over the glass and MoO_3_/glass shows green-blue and yellow colour, respectively (see photos in the inset of Fig. 6(b) and (c) respectively), which indicated the presence of an LSPR. Mondal et al. [58] demonstrated the use of MAuM in the FTO/TiO_2_/ WO_3_/CsPbIBr_2_/CuSCN/MoOx/Au/MoOx STPSCs device structure, which delivered PCE and AVT of 10.40 and 38.57%, respectively. The MAuM multilayer top electrode can also be implemented in tandem perovskite Cu(In, Ga)(Se, S)_2_ (CIGS) four-terminal solar cells [59, 60]. A simulated study conducted by Tesfancheal et al. [61] suggested that the thickness of individual layers in the DMD structure of MoO_3_/Ag/WO_3_ (MAgW) is essential in mainting PCE and AVT of STPSCs. The study suggest the ideal thickness for WO_3_ and Ag was 38 and 9 nm, respectively, to achive optimised performace using MAgW structure. Liang et al. [62] introduced the unique combination of MoO_3_/Ag/WO_x_ (MAgW) as a TTE in which the MoO_3_ enables the smoothly grown Ag film. At the same time, the top WO_x_ worked as a high-refractive-index capping layer, modulating the optical interference, helping to increase the transmittance. Figure 6(d) shows the transmittance spectra of MAgW, MAgM, and control opaque devices, between 400 and 1200 nm wavelengths. The MAgW-based device had the highest AVT (21.56%) compared to MAgM (17.63%), and both showed very high transparency in the infrared region. Moreover, the MAgW electrode-based device delivered higher PCE (up to 15.4%) than MAgM electrode (~ 14.22%). In 2019, Giuliano et al. [63] developed a DMD transparent electrode based on a MoO_x_(6 nm)/Au-seed(1.5 nm)/Cu(9.5 nm)/MoO_x_(20 nm) stack via thermal evaporation, and it is used in an FTO/c-TiO_2_/CH_3_NH_3_PbI_3 − x_Cl_x_/Spiro-OMeTAD/electrode device structure. The Cu-based DMD electrode has high conductivity and transparency. However, Cu diffuses into MoO_x_ film during the deposition process and causes a detrimental effect on conductivity and transparency. Figure 6(e) displays that from 380 to 1020 nm wavelength, the gold-seeded Cu layer (1.5 nm Au + 9.5 nm Cu) exhibited better transmittance than the pure Cu (11 nm) film. The findings imply that development a continuous film efficiently suppresses the Rayleigh-scattering and absorption properties of LSPR in the nanosized Cu clusters, resulting in improved transparency. The major transformation in the morphology of the Cu film is solely responsible for the conductivity improvement. An Au seed layer of 1.5 nm acted as a barrier to Cu deposition and limited the diffusion. The device exhibited a maximum PCE of 12.5%, with corresponding 5% AVT in the range of 400–800 nm. Figure 6(f) shows the aging effect of MoO_x_/Au(11 nm)/MoO_x_, and MoO_x_/Au-seed/Cu/MoO_x_ top electrode-based STPSCs. After 500 h storage in an N_2_-filled glovebox, both device’s PCE degraded by 70% of their initial values. The inherent ageing processes, mostly due to the perovskite degradation, are not affected by the substitution of Au for Cu. Wu et al. simulated Au-Ag, Cu-Au, and Ag-Cu bilayer metal films based DMD structure as a TTE for STPSCs [64]. The structure includes the MoO_3_/Ag/Au/ MoO_3,_ MoO_3_/Au/Cu/ MoO_3,_ and MoO_3_/Ag/Cu/ MoO_3_ DMD as a TTEs, and simulation also investigated the impact of varying thickness combinations of different metal thin films. The Ag/Cu combination of 9 /1 nm thickness enhanced the STPSCs device performace. The multilayer TTE provides high transmittance and low resistance, and contributed to improving the AVT and PCE of STPSCs [65]. Meanwhile, the multilayer metal film’s DMD structure raised the concern for commercialization as it required multiple-layer deposition with precise thickness and would increase electrode cost. The DMD structure, consisted of BCP (8 nm)/Ag (20 nm)/MoO_3_ (5 nm) has been demonstrated by Sharma et al. [66], In the FTO/NiOx/FBASMs/perovskite/PCBM/DMD device structure, the PCE and AVT delivered up to 15.12% and 30%, respectively. The NiO/Ag/NiO (NAgN) material structure as a top electrode for STPSCs also been suggested at the optimize thickness of 37 nm, 9 nm, 34 nm for NiO, Ag, and NiO, respectively [67]. The author also suggested replacing spiro-OMeTAD by NiO would improve the device stability and reduce the cost.

**Table 1:** Examples of performance of STPSCs using different photoactive layers and top electrode materials

Top electrode materials	Device structures	PCE (%)	Transmission (%)	Ref.
Metal and metal nanowires	ITO/PEDOT: PSS/Perovskite/PCBM/PEI/Ag NW (inkjet)	14.17	21.2 (AVT)	[31]
	FTO/c-TiO_2_/Perovskite/spiro-OMeTAD/Au (10 nm)	3.5	30 (AVT)	[33]
	ITO/PEDOT: PSS/Perovskite/Au (6 nm)/LiF (100 nm)	6.4	29 (AVT)	[34]
	ITO/PEDOT: PSS/Perovskite/PCBM/BCP/Ag (10 nm)/V_2_O_5_ (20 nm)	14.62	-	[35]
	ITO/PEDOT: PSS/Perovskite/PC_60_BM/ZnO/Ag NW (doctor-blading)	8.49	28.4 (AVT)	[37]
	FTO/TiO2/Perovskite/Spiro-MeOTAD/Ag NWs (spray-coated)	6.47	~ 15, at 650 nm	[42]
	ITO/PEDOT: PSS/ Perovskite/PCBM/PEI/Ag NW (spray-coated)/ZnO	7.3	23.3 (AVT)	[38]
Graphene and carbon nanotubes	PET/TETA-Graphene/ZnO/ perovskite/PTAA/PEDOT: PSS/ TFSA- Graphene/PET	10.56	5 (AVT)	[39]
	FTO/TiO_2_/MAPbI_3_/SWCNTs	10	-	[54]
Dielectric/Metal/Dielectric	FTO/TiO_2_/perovskite/spiro-OMeTAD/MoO_3_ (20 nm)/Au (10 nm)/MoO_3_ (35 nm)	5.3	31 (AVT)	[57]
	ITO/SnO_2_/Perovskite/Spiro-OMe-TAD/MoO_3_ (30 nm)/Ag (12 nm)/WO_3_ (30 nm)	15.4	21.54 (AVT)	[62]
	FTO/SnO_2_/C_60_-SAM/Perovskite/poly-VNPB/MoO_x_ (10 nm)/Au (10 nm)/MoO_x_ (10 nm)	13.7	11.7	[60]
	FTO/NiOx/FBASMs/Perovskite/(bathocuproine)/Ag(20 nm)/MoO_3_(5 nm)	15.12	30 (AVT)	[66]
Metal oxides	ITO/NiO/Perovskite/PCBM/ZnO/ InZnSnO (IZTO) (200 nm)	9.08	22.17	[45]
	FTO/c-TiO_2_/m-TiO_3_/Perovskite/Spiro-OMeTAD/MoO_x_ (10 nm)/ indium zinc oxide (IZO) (120 nm)	10.36	-	[43]
	ITO/NiOx/Perovskite/PMMA: PCBM/ZnO/InZnSnO (IZTO)	13.61	24.7 (AVT)	[46]
	ITO/MeO-2PACz/ Perovskite/ C60 /BCP + Ag (1 nm) /IZO (180 nm)	16.14	-	[44]

### Conducting polymers

**Fig. 7:**
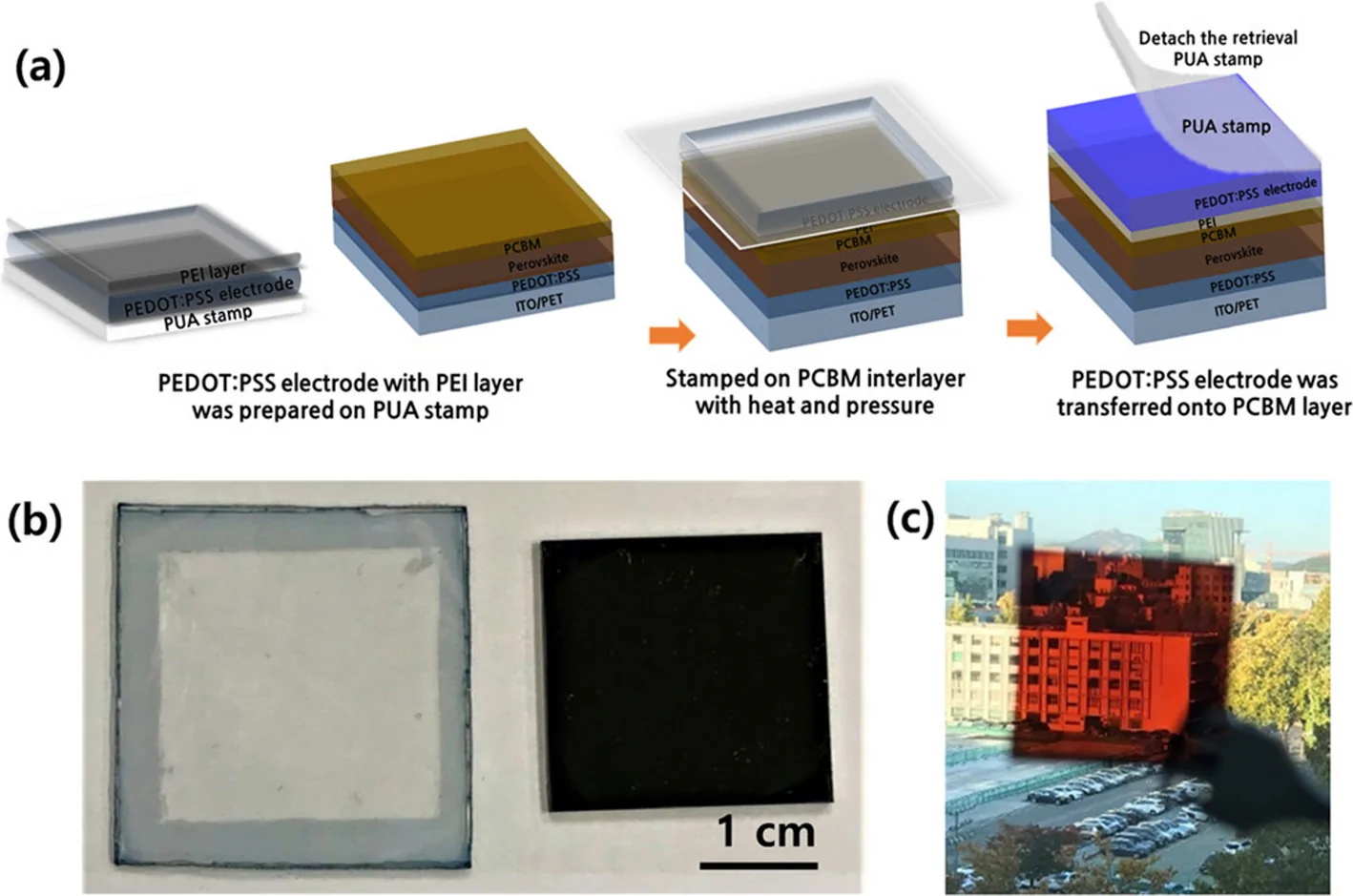
(**a**) Illustration of dry stamping transfer process, (**b**) transferred top electrode (PEDOT: PSS), and (**c**) fabricated flexible STPSCs photograph. Reproduced with permission. Copyright © 2020 American Chemical Society [77].

In the past few years, conducting polymers have emerged as strong candidates for use as top electrode materials in STPSCs due to their excellent mechanical flexibility, outstanding optical transparency and electrical conductivity [68]. Polymers such as PEDOT: PSS exhibit remarkable transparency in the visible spectrum, making them suitable for applications where light penetration is critical, such as in tandem solar cells and building-integrated photovoltaics [69]. These polymers are available in liquid form, making them cost-effective and suitable for low-temperature processing on flexible substrates [70–72]. They also form smooth, conformal contacts with the perovskite layer, enhancing the interface quality. Their tunable work function also enables optimal energy level alignment, improving device performance and long-term stability [73, 74]. Jiang et al. [75] investigated PEDOT: PSS as a top electrode material in STPSCs, replacing conventional metal electrodes. They reported a maximum PCE of 11.3% under AM1.5 illumination. The study highlighted that using the polymer enabled a simpler device fabrication process while offering high transparency and conductivity. In 2019, Zhang et al. [76] used PEDOT: PSS as an electrode in STPSCs using vacuum free lamination process by changing the transparencies to produce the most efficient device that shows the PSCs efficiency up to 16.4%. Lee et al. [77] presented the dry stamping transfer method to coat PEDOT: PSS (PH1000) as a TTE for flexible STPSCs in the following device structure, PET/ITO/ PEDOT: PSS/ FAPbI_3 − x_Br_x_/PCBM. Spin coating of PEDOT: PSS was not possible on top of perovskite layer as it contains water and can decompose the layer underneath. The author applied the dry stamping transfer method for depositing polymer top electrode, as mentioned in Fig. 7a. The transferred top electrode picture using dry stamping method and final device image are shown in Fig. 7(b-c).

## Cost, environmental impact, and commercial viability considerations

While significant progress has been made in developing high-performance materials for STPSCs, practical deployment requires careful cost evaluation, environmental impact, and industry readiness. Though widely used for their excellent optoelectronics properties, indium-based materials such as ITO suffer from high raw material costs and resource scarcity, which hinder scalability [78]. It has driven the research community toward indium-free alternatives like AgNWs, conductive polymers, carbon-based materials, and MXenes. Many of these materials, especially AgNWs and carbon allotropes, are earth-abundant and compatible with low-temperature solution processing, reducing fabrication costs and energy consumption [78]. Environmental considerations are particularly critical in the case of lead-based perovskites. Although lead contributes to high efficiency and defect tolerance, its toxicity poses serious risks during manufacturing, usage, and disposal [79, 80]. To mitigate this, various encapsulation strategies and lead-sequestering interlayers are being developed. Furthermore, lead-free perovskite alternatives, such as tin-based or double perovskites (e.g., Cs₂AgBiBr₆), are actively being explored, though they currently face limitations in efficiency and stability [81]. From an industry perspective, in the past few years an interest has grown in using STSCs for BIPV, greenhouses, agrivoltaics, and smart windows. Companies like Saule Technologies and Ubiquitous Energy have launched prototypes and pilot products demonstrating STPSCs modules. These developments underline the market potential of STPSCs, particularly where aesthetics and light transmission are valued alongside energy generation. However, scalability will depend on advances in environmentally benign, cost-effective material systems and robust encapsulation techniques. Therefore, ongoing efforts must align high-performance material innovation with lifecycle sustainability, cost reduction, and commercial feasibility to ensure the large-scale adoption of STPSC technologies.

## Challenges and future prospects

Substantial efforts have indeed been invested in producing STPSCs, and various components and approaches have been used to boost the devices’ stability and performance. The development of STPSCs requires several diverse tactics, including modification of interface materials, device structure, transparent electrodes, and transparent perovskite layers. However, the deposition of the transparent electrode is the most crucial step in the device fabrication process. Regarding applications, it is essential to consider the performance, colour, and transmittance of the STPSCs. These are the three most crucial criteria. For instance, colour-neutral STPSCs function very well for applications involving curtain walls and windows, while coloured devices are often employed for aesthetic purposes. In order to produce STPSCs with a variety of colours, the standard approach involves optimising the active layers by modifying the thickness or adjusting the bandgap of the perovskite layer. The TTEs that were utilising metal thin film and conductive oxides are now frequently employed to construct STPSCs, even though severe and costlier deposition processes restrict the manufacturing of large-area devices. In addition, multilayer metallic thin film-based TTEs, like DMD, are challenging because it is difficult to regulate these electrodes’ contending transmittance and conductivity properties. It has been discovered that metallic thin film-based electrodes incorporated with dielectric materials have significantly low transmittance. As a result, it is believed that two distinct materials with a comparable refractive index may be utilised as dielectric, and the thickness of the metal sandwiched between two distinct dielectric materials must be strictly controlled. Deposition of an thin metal film in the multi-layered electrodes is the only way to achieve high transmittance. However, this daunting challenge has led to a drop in conductance and transmittance due to metal islands forming during sputtering or vapour deposition. In order to make a homogeneous surface with fine grains that have better conductivity and transmittance, it is necessary to have procedures that halt the coarsening of the grain caused by the creation of islands in the thin metal film during the deposition process. The transparent metal oxide-based electrodes and metal oxides doped with abundant materials like Al can significantly decrease the cost of the material as compared to the scarce indium in ITO. On the other hand, using highly-priced deposition techniques like atomic layer deposition or sputtering generates a constraint for large-scale device fabrication. The mechanical characteristics of doped metal oxides do not lend themselves well and are incompatible with the scale-up roll-to-roll fabrication approach.

The solution phase deposition techniques for TTEs are needed to build flexible and large-area STPSCs via roll-to-roll technology. The STPSCs fabricated using TTEs nanomaterials such as CNT, Ag NWs, PEDOT: PSS, and graphene have the flexibility of solution deposition and may also be employed in flexible STPSCs. Despite the benefits of using these nanomaterials as TTEs, the deposition processes remain a great deal of space for improvement in optimising various characteristics. For example, while Ag NW films exhibit good conductivity and transmittance, their weak durability when subjected to continuous light exposure and a moisture-laden environment is a drawback. In addition, the Ag NW film had weak adherence with the lower-lying interfacial layers in STPSCs, which hindered its utility in the fabrication of devices on a wide scale. In order to achieve stronger adhesion, it is necessary to embed Ag NWs with functional groups that have a better capability for bonding with the interfacial layers. Electro-migration of Ag atoms was found to cause the breakdown of nanowire networks in solar cells, which endure significant operating current flow. Similarly, CNTs and graphene exhibit robust conductivity, yet their utilisation is constrained by the roughness of the films formed as TTEs. Graphene produced through solution processes such as chemical reduction or exfoliation is susceptible to various flaws, constituting a second challenge associated with this material. The presence of flaws in graphene adversely affects the conductivity of the films. In the case of electrodes made from CNTs, the limited mechanical flexibility of the device poses a significant issue. While PEDOT: PSS films demonstrate favourable optical properties, their shelf life and acidic nature may compromise long-term device stability [82, 83]. The Ag NW-based electrodes are the best-suited TTEs, and considerable research has been done on fabricating STPSCs utilising Ag NW. It can be processed as a solution. However, considering Ag NW films’ stability, further work must be done to create a stable, transparent electrode by integrating Ag NWs with other materials. For boosting the electrode’s stability and optoelectronic features, the electrode made by producing a composite of Ag NWs and other materials, such as CNTs, PEDOT: PSS, and graphene, would be the best alternative. The composite electrodes keep their smooth surface and have a high contact area with the active layer’s underneath. To produce STPSCs, in addition to the composite electrodes based on Ag NW, an electrode that has been constructed by producing a composite of other materials, such as a thin metal film with graphene and PEDOT: PSS, may also offer a high-quality TTE. The alignment of the Ag NWs in the film plays an important part in delivering consistent and exceptional conductivity throughout the film. This alignment of the Ag NWs in the film may be accomplished using the capillary printing technique. In order to create Ag NW-based TTEs, a hot-pressing deposition method and capillary printing process need to be employed in the future. According to the findings presented in this analysis, the materials that may be used to fabricate transparent electrodes are restricted to TCOs, CNTs, graphene, PEDOT: PSS, Ag NWs, and metal thin films.

Emerging materials such as MXenes, doped polymers, and carbon–metal–polymer hybrids are opening new avenues in TTE design. MXenes, especially Ti₃C₂Tₓ and its functionalized variants, offer a compelling blend of high electrical conductivity, mechanical flexibility, tunable work functions, and chemical compatibility with perovskite layers [84, 85].

MXenes have already introduced in perovskite solar cells as interfacial modifiers and additives in charge transport layer, which enhanced efficiency [86]. However, a few challenges exist, such as low intrinsic conductivity and oxidation sensitivity [87]. Several actionable research directions must be pursued to advance the practical implementation of MXene-based TTEs in STPSCs. Firstly, the development of oxidation-resistant MXene formulations or effective encapsulation strategies is critical to ensure long-term environmental stability. Additionally, exploring synergistic effects in MXene-based hybrid electrodes, such as MXene–Ag nanowire (Ag NW) and MXene–graphene systems, can potentially combine high optical transparency with excellent charge carrier mobility. Emphasis should also be placed on designing low-temperature, solution-processable deposition techniques, including spray coating and inkjet printing, to enable compatibility with flexible substrates and scalable roll-to-roll manufacturing. Moreover, interfacial engineering strategies, such as the incorporation of self-assembled monolayers or interfacial dipole layers, can enhance energy level alignment and suppress carrier recombination at the TTE–perovskite interface. Finally, establishing standardised protocols to assess the mechanical, environmental, and operational stability of these novel TTE configurations under realistic conditions will be essential for their successful commercialisation [88, 89]. 

## Conclusions

In this review, we have emphasised the pivotal role of top electrodes in advancing STPSCs, discussing in detail their critical parameters, deposition techniques, recent material developments, existing challenges, and prospects. Developing efficient TTE is essential for integrating STPSCs into various applications, including architectural glass, smart windows, foldable curtains, and energy-harvesting surfaces in homes, commercial buildings, and wearable electronics. Traditional metallic top electrodes such as Al, Ag, and Au,particularly in ultrathin layers (10–20 nm) have demonstrated high conductivity but suffer from limited optical transparency, hindering their effectiveness in STPSCs. To address this, DMD multilayer architectures, where ultrathin metal layers (< 12 nm) are sandwiched between high-refractive-index dielectric materials, have shown potential to balance transparency and conductivity. However, fabricating such electrodes often involves costly vacuum deposition techniques, limiting their scalability and commercial viability. To overcome these challenges, researchers are increasingly exploring low-cost, solution-processable alternatives, including CNTs, conducting polymers, graphene, AgNWs, and, more recently, two-dimensional materials like MXenes. Owing to their high electrical conductivity, tunable work function, mechanical flexibility, and excellent optical transparency, have emerged as promising candidates for transparent electrode applications in STPSCs. Moreover, their compatibility with scalable, low-temperature processing makes them attractive for roll-to-roll manufacturing and flexible device integration. Despite these advancements, cost remains a critical barrier to commercialisation. While perovskite materials themselves are relatively inexpensive and offer high PCE, the challenge lies in developing scalable, stable, and economically viable top electrode materials and fabrication techniques. The increasing global demand for clean and space-efficient energy solutions underscores the urgency for continued interdisciplinary collaboration between academia and industry. By leveraging the unique optoelectronic properties of perovskites along with innovative electrode materials like MXenes, the path forward for STPSCs lies in the realisation of low-cost, high-performance, and multifunctional solar technologies that can seamlessly integrate into the built environment and wearable electronics of the future.

## Data Availability

No datasets were generated or analysed during the current study.
